# Forms, Crosstalks, and the Role of Phospholipid Biosynthesis in Autophagy

**DOI:** 10.1155/2012/931956

**Published:** 2012-01-16

**Authors:** Leanne Pereira, John Paul Girardi, Marica Bakovic

**Affiliations:** ^1^Department of Human Health and Nutritional Sciences, University of Guelph, 50 Stone Road East, Guelph, ON, Canada N1G 2W1; ^2^Department of Human Health and Nutritional Sciences, University of Guelph, Animal Science and Nutrition Building, Room 346, Guelph, ON, Canada N1G 2W1

## Abstract

Autophagy is a highly conserved cellular process occurring during periods of stress to ensure a cell's survival by recycling cytosolic constituents and making products that can be used in energy generation and other essential processes. Three major forms of autophagy exist according to the specific mechanism through which cytoplasmic material is transported to a lysosome. Chaperone-mediated autophagy is a highly selective form of autophagy that delivers specific proteins for lysosomal degradation. Microautophagy is a less selective form of autophagy that occurs through lysosomal membrane invaginations, forming tubes and directly engulfing cytoplasm. Finally, macroautophagy involves formation of new membrane bilayers (autophagosomes) that engulf cytosolic material and deliver it to lysosomes. This review provides new insights on the crosstalks between different forms of autophagy and the significance of bilayer-forming phospholipid synthesis in autophagosomal membrane formation.

## 1. Introduction

Eukaryotic cells have evolved numerous pathways to improve survival in harsh environments. One such pathway, known as autophagy, specializes in the breakdown of cell components through specific and nonspecific delivering to the lysosome. The products of lysosomal degradation can then be used for the biosynthesis of new proteins and organelles and as an energy source [1]. To date, three forms of autophagy have been identified and characterized. This review will discuss key findings in autophagy research as well as provide new insights on the role of membrane lipids in autophagosome formation.

Three major forms of autophagy have been identified in cells: chaperone-mediated autophagy (CMA), microautophagy (MiA), and macroautophagy (MaA). CMA is a selective protein delivering system which uses specific heat shock protein complexes (HSPC) to deliver proteins to the lysosome for degradation [2]. CMA is unique in that it specializes in the sequestration and degradation of a single-protein substrate, whereas both MiA and MaA specialize in bulk sequestration and degradation of cytosolic components. MiA is characterized by the engulfment of cytoplasm (including proteins and organelles) by membrane invagination of lysosome and/or endosome in mammals, or vacuole in yeast [3]. Finally, MaA is distinguished by the formation of a specialized double-membrane vesicle termed the autophagosome, which forms around the material to be digested (organelles/proteins). Once the autophagosome is formed, it fuses with a lysosome forming an autolysosome [4]. Though similar in their means of cargo degradation, the three forms differ in the manner that they use to deliver their cargo to the lysosome.

To date, most autophagy research has been directed towards MaA, resulting in large gaps in our understanding of CMA and MiA processes. However this does not mean that these processes are any less important than MaA, as numerous studies have identified an association between various diseases (Parkinson's disease, Alzheimer's disease, type-II diabetes, obesity, cardiovascular disease, and cancer) and generally unregulated or defective autophagy processes [5–12]. And, as will be shown in this review, surmounting evidence suggests that there is considerable crosstalk between these three pathways.

## 2. Chaperone-Mediated Autophagy

**Figure 1:**
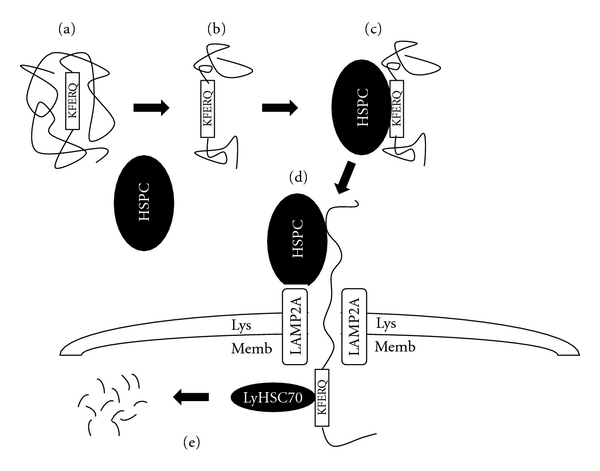
Chaperone-mediated autophagy. The KFERQ sequence of the substrate protein is protected by its three-dimensional structure, preventing its interaction with the CMA heat shock protein complex (HSPC) (a). Unfolding or improper folding of the protein (b) allows the HSPC to bind to the protein (c), targeting the protein to the lysosome for degradation (d). The CMA-specific protein is unravelled at the lysosomal membrane by HSPC and transported into the lysosome via a channel formed from LAMP2A. Lysosomal Hsc70 (ly-Hsc70) pulls the CMA specific protein into the lumen of the lysosome, where it is degraded.

CMA is a specific form of autophagy targeting only soluble proteins for delivery to the lysosome. In order for a protein to be degraded, its specific motif KFERQ is initially recognized by a large heat shock protein complex (HSPC, Figure 1). The HSPC is made from three heat shock proteins (Hsc70, Hsp40, and Hsp90), Hsc70-interacting protein (Hip), Hsc70-Hsp90-organizing protein (Hop), and Bcl2-associated athanogene 1 protein (BAG-1) (Figures 1(a)–1(c)). Within the complex, Hsc70 specifically recognizes the KFERQ motif of the substrate protein. The interaction between Hsc70 and the substrate protein is controlled by ATP hydrolysis where the ADP-bound form of Hsc70 has the greatest affinity for the substrate [13]. The other components of the HSPC complex act as cochaperones regulating the activity of Hsc70. The ADP bound form of Hsc70 then targets the HSPC to the lysosomal membrane. Transport of the protein substrate into the lumen of the lysosome requires a transmembrane protein called the lysosome-associated membrane protein 2A (LAMP2A), which acts as a receptor for substrate proteins and has been proposed to be a rate limiting step in the CMA pathway [13–15] (Figure 1). LAMP2A is one of three splice variants of the LAMP2 gene. It contains a large heavily glycosylated portion within the lumen, a ~20 amino acid transmembrane component, and ~12 amino acid tail on the cytosolic side of the lysosome [16]. The three splice variants contain a similar luminal and transmembrane portion and differ in their cytosolic tails. The short cytoplasmic tail of LAMP2A contains four positive amino acids (KRHH/KHHH) specifically required for the translocation of the substrate protein into the lysosome [17]. Once the substrate binds to the LAMP2A monomer, a 700 kDa LAMP2A complex forms, and it, as well as a luminal chaperone (lys-Hsc70) aid in protein translocation into the lumen of the lysosome, where it is degraded [14, 18]. Importantly, before the protein can be transported into the lysosomal lumen, it must first be unfolded at the lysosomal surface (Figure 1(d)).

## 3. Chaperone-Mediated Autophagy Regulation

Generally speaking, CMA regulation is poorly understood, but it is acknowledged that levels of LAMP2A are controlled mainly through its assembly and disassembly within the lysosome itself, and not through gene expression within mammalian cells. During disassembly, LAMP2A is first truncated before entering the lumen where it is degraded. During nutrient deprivation, degradation rates of LAMP2A are reduced and remain so for a longer duration at the lysosomal membrane, allowing CMA to occur at greater rates [17]. Hsc70 has recently been shown to play a major role in the disassembly of the LAMP2A complex, whereas luminal Hsp90 stabilizes LAMP2A at the lysosome membrane [18] and the lysosomes which contain a higher amount of lys-Hsc70 seemingly have more active CMA [19].

## 4. Microautophagy

**Figure 2:**
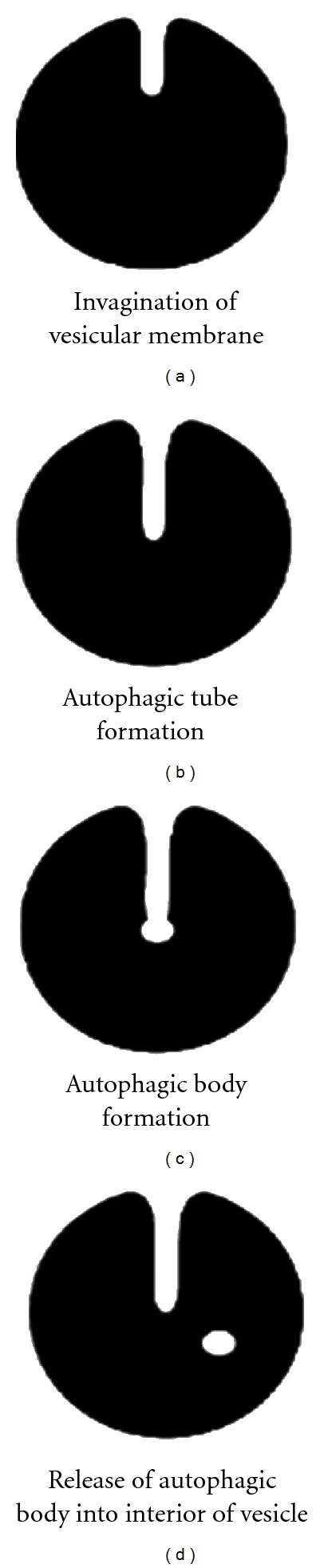
Microautophagy. A direct invagination of a vesicular membrane (lysosome and/or endosome) takes in cytoplasm from the surrounding environment (a). This invagination continues and forms a narrow tube which elongates into the interior of the vacuole (b). This tube is referred to as the autophagic tube and is continuous with the cytoplasm. Entire organelles or compounds within the taken up cytoplasm are degraded within the lumen of the vacuole (if taken in via the lysosome) or fusion with a lysosome occurs, which results in the breakdown of the captured cytosolic components. As the tube reaches the interior of the vacuole, the proximal ends form a bulge referred to as the autophagic body (c). This bulging end is thicker than the tube leading into the vacuole. The walls of the bulging end of the autophagic tube will then fuse, pinching off into the inner portions of the lysosome (d). The cytosolic components trapped within the vesicle are then degraded within the vesicle (lysosome or vacuole) or the vesicle (endosome) will later fuse with a lysosome, where the components are then degraded.

In MiA, a direct invagination of a specialized vesicular membrane takes in cytoplasm from the surrounding environment (Figure 2). This invagination grows and forms a narrow tube which elongates into the interior (Figure 2(a)). This tube is referred to as the autophagic tube and is continuous with the cytoplasm (Figure 2(b)). As the tube reaches the interior of the vesicle, the proximal membrane ends form a bulge, referred to as the autophagic body (Figure 2(c)). This bulging end is thicker than the tube leading into the interior. The walls of the bulging end of the autophagic tube then fuse, pinching off into the inner portions of the lysosome (Figure 2(d)). The cytosolic components trapped within the body are degraded within the vacuole (lysosome) or the vacuole will later fuse with a lysosome and the components degraded [3].

Similar to CMA, the processes involved in the regulation of MiA still need to be elucidated. However within yeast, studies have shown that within the later stages of MiA the pinching of the vesicle at the interior end of the autophagic tubule is controlled through a vacuolar transporter cochaperone (VTC) complex, a protein which is localized at the endoplasmic reticulum (ER) and on vacuolar membranes. Although the autophagic tubule was produced, the pinching off of the vesicle from the tube was impaired when VTC complex formation was prevented [3]. It also appears that calmodulin plays an important role during invagination of the autophagic tubule. Although this interaction is calcium independent, VTC has been identified as a direct target of calmodulin, strengthening the role of VTC during MiA [3, 20].

## 5. Nuclear or Piecemeal Microautophagy

**Figure 3:**
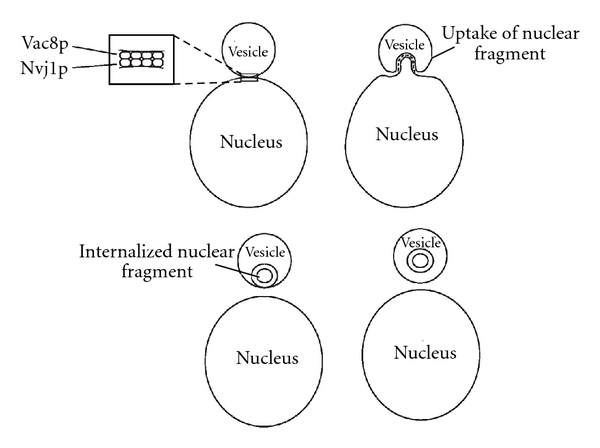
Piecemeal Microautophagy. In yeast, the vacuole and nuclear membrane form a tight junction between proteins Vac8p (Vacuole) and Nvj1p (Nuclear) (a). As the vacuole invaginates, the nuclear membrane is pulled inward due to the interaction between Vac8p and Nvj1p (b). This continues until an autophagic-like body forms within the vacuole and pinches off from the membrane (c) and is released into the vacuole where it is later degraded (d). Vac8p: Vacuolar 8 protein; Nvj1p: nucleus vacuole junction 1 protein.

Extensive research in yeast has identified a form of autophagy in which portions of the nucleus undergo degradation through a MiA-like process. The outer nuclear membrane interacts with the vacuole through a nucleus-vacuole junction mediated by the nucleus vacuole junction 1 protein (Nvj1p) localized on the outer nuclear membrane and vacuolar 8 protein (Vac8p) found on the vacuolar membrane [21] (Figure 3). After the contact, the vesicle invaginates inward pulling on the outer membrane of the nucleus and the components into the autophagic body where they are later released into the vacuole [22] (Figure 3). After upregulation of piecemeal MiA with rapamycin, an increased presence of the chaperone VTC was observed at the nuclear-vacuole junction, suggesting a specific role of VTC in this process [23]. Interestingly, the nuclear protein Nvj1p is also responsible for the recruitment of Tsc13, a protein which plays a role in the synthesis of very-long-chain fatty acids, and Osh1, a protein responsible for nonvesicular lipid transport. The recruitment of Tsc13 and Osh1 in MiA suggests that additional lipids may be required for the formation of the inner membranes forming within the vacuole [24]. Finally, similar to CMA, MiA and MaA also occur at similar times during certain stimuli (nutrient deprivation) and the proteins specific for MaA are actively involved in piecemeal MiA. Autophagy-related proteins, Atg3, Atg7, Atg12, Atg16, Atg1, and VPS (vacuolar protein sorting) 30, and many other proteins which play key roles in MaA were shown to play active roles in piecemeal MiA [25].

## 6. Endosomal Microautophagy

There has been a selective and nonselective form of endosomal MiA identified [26]. In the selective form (similar to CMA, Figure 1) proteins containing the KFERQ sequence were targeted for degradation after binding to Hsc70. LAMP2A is not localized on the late endosomes (LE), suggesting that Hsc70 is interacting with the LE membrane through a mechanism unlike CMA during the delivery of specific proteins (KFERQ sequence) for degradation. Interestingly, Hsc70 has a preference for interacting with regions of membrane containing acidic phospholipids (phosphatidylserine, phosphatidylinositol, and phosphatidylglycerol). When considering the composition of cellular membranes, it is likely that phosphatidylserine (PS) is largely responsible for the interaction between the LE and Hsc70 due to its ability to play a role in protein localization [27]. Additionally, the endosomal MiA relies on ESCRT 1 and 3 systems for the formation of the MiA vesicles. When ESCRT functioning was disrupted, although a thin-like vesicle (autophagic tubule) was formed within the LE, pinching off of the vesicle did not occur [26].

## 7. Microautophagy Regulation

**Figure 4:**
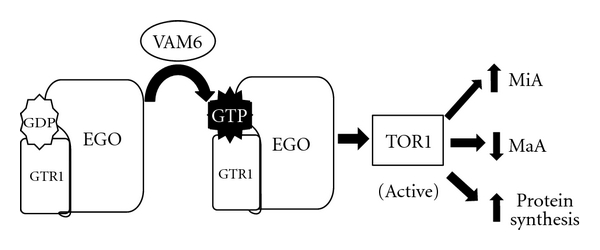
Activation of TOR1 in microautophagy. Vam6 activates Gtr1 of the EGO complex leading to the activation of TOR1. Through the activation of TOR1, microautophagy levels increase and the cell increases protein synthesis as it returns to a growing state. MaA, typically activated in nutrient deprived conditions, is inhibited.

The target of rapamycin (TOR), the central regulator in cell growth in yeast, and mammalian cells can exist in two forms, TOR1 and TOR2 (mTOR1 and mTOR2 in mammalian cells). Interestingly, it is TOR1 which, when active, decreases the rate of protein synthesis, causing an arrest in the cell cycle and mimicking the effects seen during nutrient starvation, and preventing macroautophagy from occurring. TOR is a positive regulator of microautophagy due to its relationship with the EGO complex, which is made up of EGO1, EGO2, GTR1, and GTR2 [28, 29]. TOR1 and the EGO complex colocalize with Vam6 at the vacuolar endosomal membrane [30]. In fact it is now believed that the subunits GTR1 and GTR2 of the EGO complex may play a vital role in regulating the activity of TOR1 in yeast [30, 31] (Figure 4).

Interestingly the EGO complex is also located on the vacuolar membrane in yeast and was shown to play a role in monitoring the size of the vacuole during microautophagy. Through continuous fusion of autophagosomal membranes with the vacuole, the size of the vacuole continues to increase, but as microautophagy levels increase, the internalization of the membrane of the vacuole occurs preventing enlargement. The vesicle then buds off into the lumen of the vacuole, and the high lipid content of the vesicle is degraded. In fact mutants of EGO1 and EGO2 developed larger vacuoles, suggesting that the EGO complex is unable to induce internalization of the membrane to prevent vacuolar growth [32].

## 8. Macroautophagy

**Figure 5:**
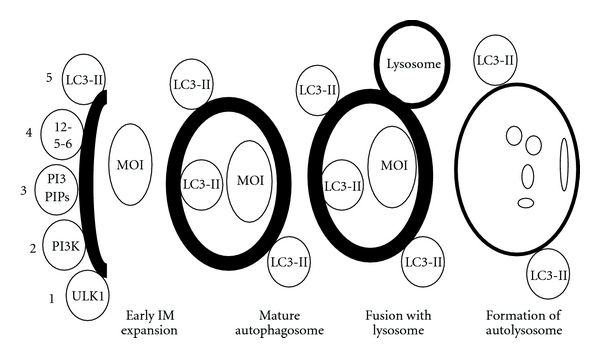
Macroautophagy. Recruitment of the large ULK1 complex to the omegasome (1) induces the migration of the class-III PI3K kinase complex to the same site (2). PI(3)PIP recruitment (3) promotes the localization of ATG12-ATG5-ATG16L (4) and LC3-II (5). The fully formed autophagosome engulfs the MOI and fuses with a lysosome, forming an autolysosome, where lysosomal hydrolases contained within the lysosome degrade the MOI. MOI: material of interest; 12-5-6: ATG12-ATG5-ATG16L; PI3P1P: phosphatidylinositol triphosphate-interacting protein; PSE: phospholipid synthesizing enzyme; LC3: microtubule-associated light chain 3; PI3K: phophoinositide 3 kinase; ULK1: unc-51-like kinase 1; ER: endoplasmic reticulum; IM: isolation membrane.

In MaA, a double-membrane structure termed an autophagosome is formed (Figure 5). This specialized vesicle delivers its engulfed material to a lysosome via membrane fusion, forming an autolysosome. Once fusion with a lysosome occurs, the components within the autolysosome are degraded (Figure 5).

Three events must occur in order to form a fully functional autophagosome in mammalian cells. The first step is the nucleation event, where recruitment of an early kinase complex is believed to trigger the synthesis of the autophagosomal isolation membrane (IM) [33, 34] (Figure 5). The specific site of autophagosome formation is believed to occur in a region of the endoplasmic reticulum (ER) called the omegasome [35]. The second step of autophagosome formation is characterized by the expansion of the IM. Finally, a fully functional autophagosome is formed through membrane fusion of the IM.

During the nucleation event, recruitment of a large serine/threonine complex consisting of autophagy-related protein 13 (ATG13), focal adhesion kinase family interacting protein (FIP200), Unc-51-like kinase 1 (ULK1), and ATG-101 translocates to the ER region of the omegasome [33, 36] (Figure 5). Translocation of this complex induces recruitment of the large-class-III phosphoinositide 3 kinase (PI3K) complex consisting of vacuolar protein sorting (VPS)-34, VPS-15, BAX interacting protein-1, Beclin, and ATG-14 to the omegasome [7, 33]. The large PI3K complex then phosphorylates phosphatidylinositol to phosphatidylinositol-3 phosphate (PI(3)P) and the PI(3)P bound proteins, WD-repeat protein interacting with phosphoinositide-2 (WIPI2) and double FYVE-containing protein 1 (DFCP1), translocate to the same region [33, 37]. WIPI2 and DFCP1 then promote the recruitment of the phospholipid carrier LC3-II (microtubule-associated light chain 3) to the isolation membrane. Since it is understood that the ATG5-ATG12-ATG16L specifies the site for LC3-I lipidation, it is possible that WIPI2 and DFCP1 may be positively effecting LC3-II formation via an interaction with ATG5-ATG12-ATG16L [38, 39]. The fully formed autophagosome then delivers the cytosolic components to a lysosome via vesicle fusion, forming an autolysosome (Figure 5). Lysosomal digestive enzymes contained within the autolysosome degrade the proteins and/or organelles, and degradation products are recycled for use in energy generation or other processes.

## 9. Macroautophagy Regulation

**Figure 6:**
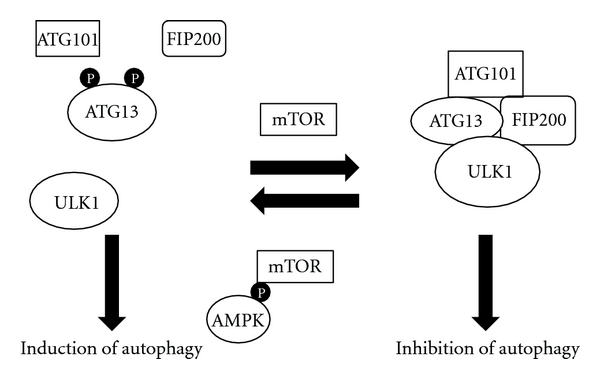
mTOR and AMPK regulation of macroautophagy. During nutrient rich conditions, mTOR remains active, phosphorylating ATG13, preventing its ability to form the large activating complex for autophagy (ATG13-FIP200-ULK1-ATG101). During periods of nutrient deprivation (amino acid deprivation, growth factor deprivation, and/or low-energy status), AMPK is activated and phosphorylates mTOR, preventing its ability to phosphorylate ATG13. This allows ATG13 to form the large activating complex, leading to the induction of autophagy. ATG: autophagy-related protein; P: phosphate; AMPK: adenosine monophosphate kinase; FIP200: focal adhesion kinase family-interacting protein of 200 kDa; ULK1: Unc-51-like kinase 1; mTOR: mammalian target of rapamycin.

The most important regulator of MaA is the mammalian target of rapamycin (mTOR), a serine/threonine protein kinase. During nutrient rich states, mTOR is active and phosphorylates ATG13, preventing its association with a large activating complex (ATG13-FIP200-ULK1-ATG101) vital for the initiation of autophagosome formation (Figure 6). When ATG13 is phosphorylated, it does not associate with FIP200, Unc 51 like kinase 1 (ULK1), and ATG101, which prevents autophagy from occurring. During periods of low-energy, amino acids and/or growth factors, mTOR is inhibited through phosphorylation of AMPK [32]. Since mTOR is a negative regulator of autophagy, its inhibition allows the formation of the large ATG13-FIP200-ULK1-ATG101 complex and it subsequently translocates to the ER to initiate the nucleation of the isolation membrane (Figure 6) [25, 35]. Translocation of the serine/threonine complex then induces recruitment of the large-class-III PI3K complex and ATG-14 to the ER [36, 40].

## 10. Crosstalk between MaA and CMA

In conditions of stress similar to which induce MaA, an upregulation in CMA results, and both processes appear to be vital to cell survival. For instance, studies in fibroblasts have shown that MaA activity rates peak within the first few hours of starvation. However, if the cell remains in this state for longer than 6 hours, CMA activity levels rise above those of MaA and reach maximal activity at about 20 hours [41]. Since MaA is a nonselective process, if its rates were to remain high, it would eventually lead to cell death due to the non-specific degradation of organelles and/or proteins required for survival and proper functioning of the cell. Through activation of CMA, the cell is able to target only nonvital proteins, allowing it to survive and function properly in a nutrient-deprived environment. Therefore it is the consecutive pairing of these two processes that maintains a cell's viability under adverse conditions. Furthermore, in situations that do not allow for CMA activity, such as the presence of large protein complexes which obstruct CMA machinery, MaA is upregulated to eliminate protein aggregates and ensure cell survival [41]. It is becoming increasing clear that there is an important link between MaA and CMA, as both pathways use similar machinery to carry out their respective actions. Moreover both pathways are upregulated by similar conditions including nutrient deprivation, oxidative stress, and/or improper protein folding in the ER. The crosstalk between CMA and MaA was most evident when MaA became upregulated in cells defective in the CMA-specific protein LAMP2A. The exact manner in which the cells compensated for decreased CMA activity is not fully understood but an overlap in proteins involved in both autophagy pathways was offered as a plausible mechanism. In the absence of LAMP2A activity, the assembly of the large CMA complex became prevented and cells had an increased availability of proteins required for CMA. If these same proteins are also required for MaA, that would lead to increase this process [42, 43].

## 11. Phospholipid Synthesis and the Formation of the Isolation Membrane

Despite the advances in autophagy research over the last few decades, one area of research still remains poorly understood. Due to the lack of lipid specific biomarkers and adequate functional assays, scientific understanding of the origin and source of the autophagosomal membranes remains incomplete. Recent evidence of the contribution of phospholipids to various processes including membrane fusion and cell survival pathways however points to a functional capacity of phospholipids outside of their normal structural role [42–45].

Phosphatidylcholine (PC), phosphatidylethanolamine (PE), and phosphatidylserine (PS) are essential bilayer forming lipids in all cells. PC is predominately found in the outer leaflet of the cell membrane and PE and PS make up the inner leaflet phospholipids [46, 47]. PE and PS have been shown to redistribute within the membrane under certain stress conditions [44, 48] where higher concentrations of PE and PS were found in the outer leaflet of the plasma membrane preceding cell-to-cell fusion of myoblasts to form myotubes [45]. Similarly, during cytokinesis, PE was found on the outer leaflet of the plasma membrane and at the cleavage furrow during late telophase [49]. These and other findings demonstrate a necessary capacity of the inner membrane phospholipids PE and PS in membrane fusion. Finally, several studies have identified a distinct function of PS in cell survival whereby localization of PS to the outer membrane leaflet of cells under stress or apoptotic conditions acts as a recognition signal for phagocytic binding [48, 50, 51].

Some of the leading hypotheses on the source of the autophagosomal membrane include the ER, the Golgi apparatus, the mitochondria, and the plasma membrane [52–55]. Increasing evidence however suggests that the ER plays a significant function in autophagosome formation by providing phospholipids for autophagosome membrane initiation and expansion. Two models propose a role for the ER in autophagosome formation. The first model suggests that the autophagosome originates from a ribosome-free region of the rough ER [56, 57]. However, a lack of certain ER markers (P450 and PDI) on the IM suggests that autophagosomes are not derived from the ER via direct maturation [35, 58–60]. In fact, IMs and mature autophagosomes were reported to appear as nascent membranes, whereby any proteins present on the membrane would have to be stripped for the maturation model to hold true [61–63]. However, protein removal from membranes is an energetically expensive process and the synthesis of new membranes from localized lipid sources is a more plausible explanation [64], which altogether fits into the second model proposing that the ER plays a fundamental role in autophagosome formation through providing newly synthesized lipids, notably the phospholipids PE, PC, and PS as described here in after.

**Figure 7:**
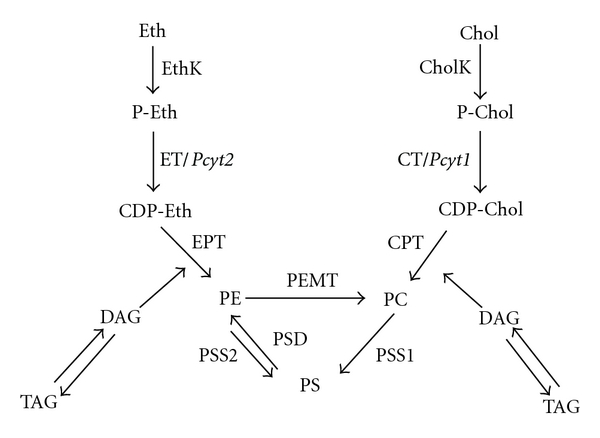
Phospholipid biosynthetic pathways. PE is made by the CDP-ethanolamine pathway in the ER (left) or from PS via the action of PSD in mitochondria. PC is made by the CDP-choline pathway (right) or from PE through the PEMT pathway in the ER and mitochondria-associated membranes of the ER. PS is produced from PE and PC via PSS2 and PSS1 pathways in the mitochondria associated membranes of the ER. Ethanolamine kinase: EthK; CTP: ethanolaminephosphate; cytidylyltransferase: ET; CDP: diacylglycerol ethanolamine and choline phosphotransferases: EPT and CPT; choline kinase: CK; CTP: cholinephosphate cytidylyltransferase: CT; CDP: diacylglycerol choline and ethanolamine phosphotransferases: CPT and EPT; Phosphatidylethanolamine N-methyltransferase: PEMT; phosphatidylserine synthase 1: PSS1; phosphatidylserine synthase 2: PSS2.

The biochemical pathways for phospholipid synthesis are described in Figure 7. The Kennedy pathway accounts for bulk synthesis of PE and PC through two independent branches of the pathway: CDP-ethanolamine and CDP-choline, respectively. In the CDP-ethanolamine pathway, ethanolamine (Etn) is phosphorylated by ethanolamine kinase (EK) to form phosphoethanolamine (Etn-P), which is then converted by CTP: phosphoethanolamine cytidyltransferase (Pcyt2/ET) into CDP-ethanolamine (CDP-Etn). Finally, PE is formed from CDP-ethanolamine and diacylglycerol (DAG) by CDP-ethanolamine: 1,2-diacylglycerol ethanolaminephosphotransferase (CEPT) [65]. Alternatively, PE can also be synthesized by the decarboxylation of PS through the action of phosphatidylserine decarboxylase (PSD) within the mitochondria [66, 67] (Figure 7 left).

Analogous to PE synthesis via the CDP-ethanolamine pathway, PC production via the CDP-choline pathway begins with the phosphorylation of choline via choline kinase (CK) to form phosphocholine (P-choline). CDP-choline is produced from phosphocholine via phosphocholine cytidyltransferase (Pcyt1/CT) and finally condensed with DAG to form PC (Figure 7 right). PC can be synthesized by two additional pathways: PE synthesized through the CDP-ethanolamine pathway can be converted to PC via the enzyme phosphatidylethanolamine N-methyltransferase (PEMT), or through the decarboxylation of PS to form PE, which can then be methylated to form PC [68–70] (Figure 7). Unlike PE and PC, which are primarily synthesized de novo, PS is synthesized largely via an exchange reaction with preexisting PE by phosphatidylserine synthase 1 (PSS1) or with preexisting PC by phosphatidylserine synthase 2 (PSS2) in mitochondria-associated regions of the ER [71]. It should be noted that while the bulk of the Kennedy pathway takes place in the cytoplasm, the final steps of PE and PC formation occur in the ER [71–77]. Similarly, the final synthesis of phosphatidylinositol (PI), a phospholipid required in the early stages of autophagosome formation, occurs at the ER through the action of phosphatidylinositol synthase [77, 78]. Though this enzyme is located in other regions of the cell, such as the plasma membrane or Golgi, it is accepted that the bulk of PI synthesis occurs at the ER [79]. Thus the ER is involved in the synthesis of all types of phospholipids (PE PC, PS, and PI) necessary for autophagosome biogenesis.

Recent studies examining the role of the Kennedy pathway in autophagy showed that when autophagy was induced by starvation in mouse hepatoma cells, activity of the rate limiting enzyme in the CDP-ethanolamine pathway, ET, increased. Furthermore, during autophagy, an increased synthesis of all membrane-forming phospholipids was seen at the level of the ER and PE produced in this pathway became incorporated into LC3 under these same conditions [64]. The ER model for autophagosome phospholipids is made even more convincing by studies conducted by J. Vance, which showed that newly synthesized phospholipids are favoured over preexisting phospholipid stores for organelle membrane production in rat hepatocytes [80]. These data were confirmed more recently by Hörl et al. who showed that lipid droplet formation requires de novo synthesis of PC in 3T3-L1 adipocytes [81].

Further evidence of the role of the ER in autophagosome formation is provided by the discovery that under starvation conditions, DFCP1 (double FYVE-containing protein 1) a PI(3)P-binding protein, translocates to the ER to form an omegasome [33]. Generation of an omegasome by DFCP1 is suggested to be necessary for the recruitment of the ATG5-ATG12-ATG16L complex, and subsequently LC3 to the omegasome [39, 82]. Moreover, more recent studies by Yla-Antilla et al. and Hayashi-Nishino et al. demonstrated that the ER associates with the IM, forming what Hayashi-Nishino et al. have deemed an ER-IM complex in mammalian cells [35, 53, 82]. In fact, electron-tomography data of ER-IM complexes have revealed a physical connection between the ER and the IM, whereby the inner and outer surfaces of the IMs were shown to be directly connected with the ER [83]. Interestingly, once the IMs matured into autophagosomes, the connection between the ER and the IMs was lost, further supporting the position that the ER is involved in the process of IM formation and elongation [35].

## 12. Conclusion

Considering the work on the forms of autophagy has increased, there is still a large gap in understanding how these processes are regulated. It now appears that the autophagosomal membrane is synthesized de novo during upregulation of MaA, although it is unknown whether synthesis of new membrane is required during MiA or CMA. Studies which prevent the proper functioning of phospholipid synthesizing enzymes will determine whether de novo synthesis of membrane is required for these forms of autophagy. Additionally, what is becoming increasingly clear is how MaA, MiA, and CMA are linked and share the same and/or similar proteins. Increased investigation of the crosstalk between these forms of autophagy will help to understand the specific roles these systems play (together and separately) in the removal of cellular material.
